# Design, Synthesis, Molecular Modelling and Anticancer Activities of New Fused Phenanthrolines

**DOI:** 10.3390/molecules25030527

**Published:** 2020-01-25

**Authors:** Cristina Maria Al Matarneh, Roxana Maria Amarandi, Anda Mihaela Craciun, Ionel I. Mangalagiu, Gheorghita Zbancioc, Ramona Danac

**Affiliations:** Faculty of Chemistry, Alexandru Ioan Cuza University of Iasi, 11 Carol I, 700506 Iasi, Romania; almatarneh.cristina@yahoo.ro (C.M.A.M.); amarandiroxana2006@yahoo.com (R.M.A.); olaru.anda@icmpp.ro (A.M.C.); ionelm@uaic.ro (I.I.M.)

**Keywords:** phenanthrolines, anticancer, tubulin docking, 3 + 2 cycloaddition, phenstatin

## Abstract

Three series of fused pyrrolophenanthroline derivatives were designed as analogues of phenstatin and synthesized in two steps starting with 1,7-phenanthroline, 4,7-phenanthroline and 1,10-phenanthroline, respectively. Two (Compounds **8a** and **11c**) of the four compounds tested against a panel of sixty human cancer cell lines of the National Cancer Institute (NCI) exhibited significant growth inhibition activity on several cell lines. Compound **11c** showed a broad spectrum in terms of antiproliferative efficacy with GI_50_ values in the range of 0.296 to 250 μM. Molecular docking studies indicated that Compounds **8a** and **11c** are accommodated in the colchicine binding site of tubulin in two different ways.

## 1. Introduction

Phenanthrolines are versatile moieties which have found use in many applications [1,2,3] due to a wide variety of interesting properties (complexation [1,2,3], luminescence [1,2,4], biological activities [4,5,6,7], semiconductors [8,9,10]). These polycyclic compounds are also naturally occurring in morphine alkaloids, sterols or hormones [11], which makes the phenanthroline scaffold an excellent choice in the generation of unique bioactive compounds.

At the same time, the fusion of two or more heterocycle rings can lead to novel classes of ligands, many with new interesting properties, including anticancer activity. For example, substituted benzo[c]phenanthroline analogues of nitidine and fagaronine showed cytotoxic properties associated with DNA intercalation and induced G2/M phases arrests [12]. Synthetic pyrido [1,8] or [1,9] phenanthroline analogues of amphimedine exhibited cytotoxic activities in various cancer cell lines [13,14]. Recently, our group also reported several fused pyrrolo [1,7] or [4,7] phenanthrolines which showed moderate antiproliferative activity [15,16,17].

Despite the great progress achieved in chemotherapy, cancer remains a leading cause of mortality worldwide [18,19]. Among the most promising strategies in cancer therapy, the discovery of compounds capable of interfering with tubulin assembly gained lots of attention in recent years, due to the role of microtubules in eukaryotic cell proliferation [20,21]. Phenstatin, one of the simplest tubulin polymerization inhibitors [22,23], acts by interacting with the colchicine binding site of tubulin and exhibits anticancer activity comparable to combretastatin A-4 [24], which is currently being investigated as a neoplastic agent in a number of clinical trials. The structural simplicity of phenstatin has inspired many researchers in designing new anticancer compounds, the recent literature being plentiful of phenstatin-derived pharmacomodulators [25,26,27,28,29].

**Figure 1:**
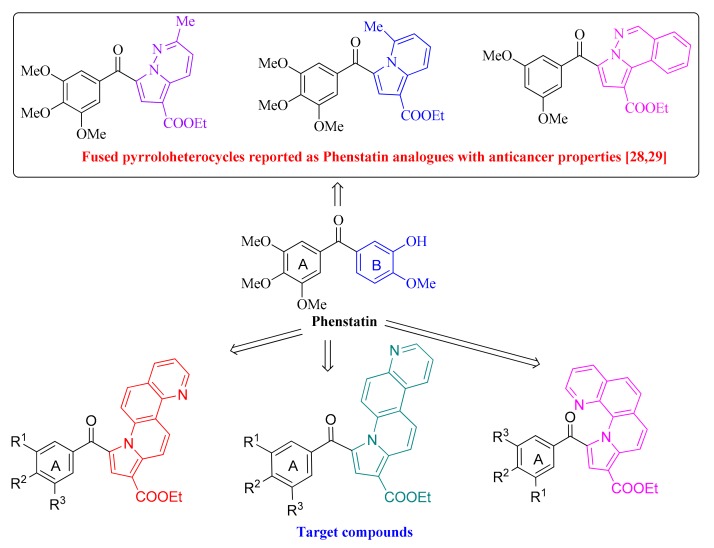
Design for the target pyrrolophenanthroline derivatives.

Encouraged by the above considerations (especially by the cytotoxic properties of the reported fused phenathrolines) and as part of our ongoing research aimed at investigating new anticancer drugs [15,16,28], we designed a new series of phenstatin analogues by replacing the 3-hydroxy-4-methoxyphenyl ring of phenstatin (ring B) with three different classes of substituted pyrrolophenanthrolines (Figure 1).

We furthermore considered some modifications at the 3,4,5-trimethoxyphenyl ring of phenstatin (ring A), generating either a 3,5-dimethoxyphenyl, 3,4-dimethoxyphenyl or a 4-bromophenyl ring.

In this way, we incorporated the trimethoxyphenyl ring of phenstatin and our fused pyrrolophenanthroline system into a single molecule, in order to investigate their impact on the anticancer activity of the parent compound. Thus, we report here the synthesis and biological evaluation of novel compounds with 1,7-, 4,7-, and 1,10-phenanthroline scaffolds.

## 2. Results and Discussion

### 2.1. Chemistry

Compounds **5a**–**d** were successfully synthesized using a two-step procedure starting from 1,7-phenanthroline **1**. The first step comprised the formation of monoquaternary 1,7-phenanthrolin-7-ium salts **3a**–**d** by the nucleophilic substitution of phenanthroline **1** to 2-bromoacetophenones **2a**–**d** (Compounds **2b**, **2c** and **2d** were obtained using reported procedures [30]).

**Scheme 1:**
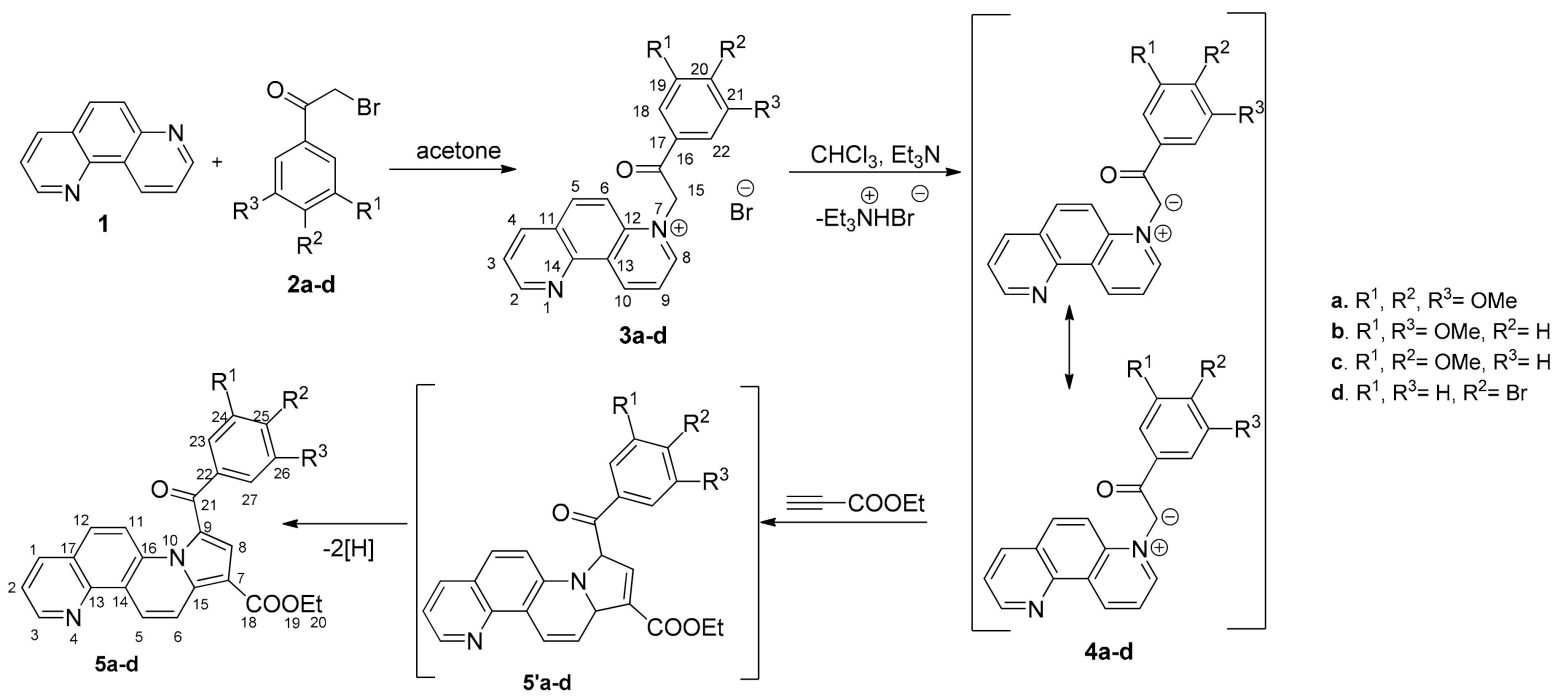
Synthesis pathway for the fused pyrrolo[1,2-*i*][1,7]phenanthroline **5a**–**d**.

The second step consisted of the in situ generation of the cycloimmonium ylides **4a**–**d** from the corresponding salts **3a**–**d** under triethylamine treatment. The in situ-formed ylides acted as 1,3-dipoles when reacted with ethyl propiolate, following a Huisgen 3 + 2 cycloaddition. Initially formed unstable intermediates **5′a**–**d** undergo an aromatization process under the current reaction conditions, leading to target compounds **5a**–**d** in 50–88% yields (Scheme 1).

**Scheme 2:**
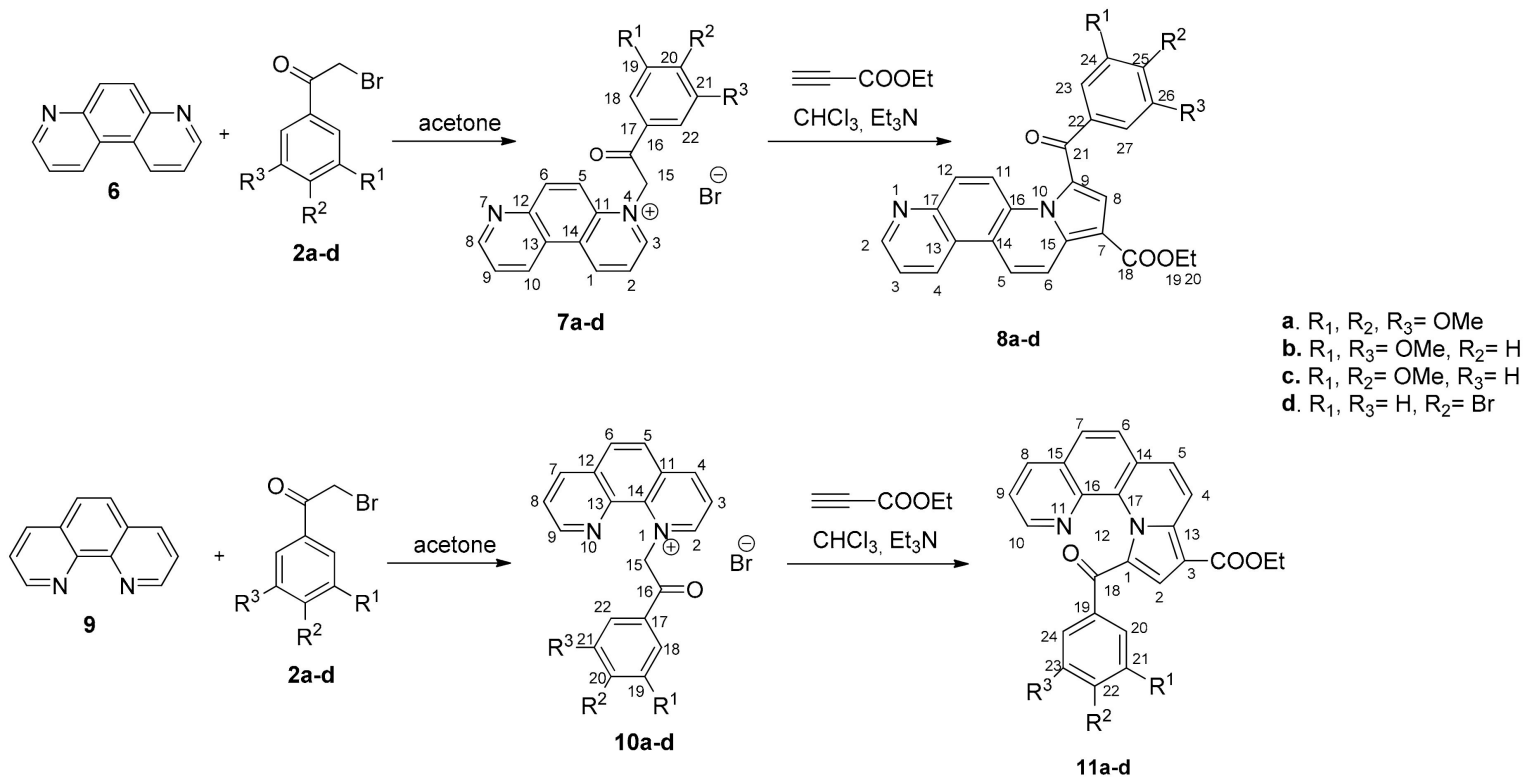
Synthesis pathways for the fused pyrrolo[2,1-*c*][4,7]phenanthroline **8a**–**d** and pyrrolo[1,2-*a*][1,10]phenanthroline **11a**–**d**.

We employed a similar strategy in the synthesis of pyrrolo[2,1-*c*][4,7]phenanthrolines **8a**–**d** and pyrrolo[1,2-*a*][1,10]phenanthrolines **11a**–**d**, by reacting phenanthrolinium monoylides (derived from 4,7- and 1,10-phenanthrolinium salts **7a**–**d** and **10a**–**d**, respectively) with ethyl propiolate (Scheme 2).

All synthesized compounds (including intermediate phenanthrolinium salts) were identified by NMR and IR. Compounds **3d**, **5d**, **10d** and **11d**, which have already been reported in the literature, showed spectral data in agreement with the reported data [16,31,32,33].

### 2.2. Anticancer Evaluation

Compounds **5a**–**d**, **8a**–**d** and **11a**–**d** were submitted to the National Cancer Institute (NCI, USA) and four compounds were selected for the evaluation of their antiproliferative activity against their panel of 60 human cancer cell lines. The NCI panel is organized into nine sub-panels representing leukemia, lung, colon, central nervous system (CNS), melanoma, ovary, kidney, breast and prostate cancer cells. The panel also includes several multidrug-resistant tumor cell lines (RXF393, HCT-15, UACC-62, SF-539). The four compounds—**5c**, **8a**, **8b** and **11c**—selected by NCI were first tested at a single high dose (10^−5^ M) in the full 60-cell panel, selected results being presented in Table 1. Compounds **8a** and **11c** showed very good growth inhibition of several cancer cell lines. The best efficacy in terms of growth inhibition was shown by Compound **11c** against the HL-60(TB) RPMI-8226 leukemia cell line, NCI-H522 non-small cell lung cancer line, COLO205 and HT-29 colon cancer cell line, SF-539 CNS cancer cell line, MDA-MB-435 and M14 melanoma cell lines, OVCAR-3 ovarian cancer cell line and A498 renal cancer cell line, these results being comparable or better than phenstatin, which was used as a control. Compound **11c** also showed cytotoxic activity against the COLO205, MDA-MB-435 and A498 cell lines. An important growth inhibition effect was also observed for Compound **8a** against MDA-MB-435 melanoma cells and K-562, SR and HL-60(TB) leukemia cells. Thus, replacement of ring B of phenstatin with a pyrrolo[1,2-*i*][1,7]phenanthroline moiety does not look favorable in terms of anticancer activity, while the replacement with a pyrrolo[2,1-*c*][4,7]phenanthroline or a pyrrolo[1,2-*a*][1,10]phenanthroline group retains the growth inhibition properties of phenstatin. The more active Compound **8a**, by comparison with **8b**, retains the three methoxy groups of the more active phenstatin. Therefore, the lack in the methoxy substituent in *para* position of the ring B appears to be not favorable for the anticancer activity, at least in the case of compounds type **8**.

Compound **11c**, which had the best growth inhibition profile among the tested compounds, progressed to the full five-dose assay. Selected GI_50_ values are presented in Table 2. The in vitro screening results revealed that Compound **11c** possess excellent to moderate antiproliferative activity with GI_50_ values ranging from 0.296 to 3.78 μM against 40 cancer cell lines from all nine sub-panels. In particular, Compound **11c** showed the best GI_50_ and TGI (the drug concentration resulting in total growing inhibition) values (296 nM and 981 nM respectively) against MDA-MB-435 melanoma cells. Promising GI_50_ values were also obtained for against NCI-H522 lung cancer cells, HCT-116 colon cancer cells and M14 melanoma cells. However, when comparing to control phenstatin, there are only a few cell lines against which Compound **11c** is more potent (HT29 colon cancer cells, SK-MEL-28 melanoma cells and T-47D breast cancer cells) or shows similar antiproliferative activity (MDA-MB-468 breast cancer cells and A498 renal cancer cells).

### 2.3. Molecular Modeling

In order to verify if our target phenstatin analogues retain the ability of their parent compound to fit in the colchicine binding site of tubulin, docking studies were performed on the four NCI-tested compounds in the colchicine binding site of the α,β-tubulin heterodimer (PDB:1SA0). The in silico study aimed to evaluate the shape and electrostatic complementarity between the tested ligands and the α,β-tubulin heterodimer interface, which could account for the observed antiproliferative effects in the case of **8a** and **11c** and the lack of activity in the case of **5c** and **8b**.

**Figure 2:**
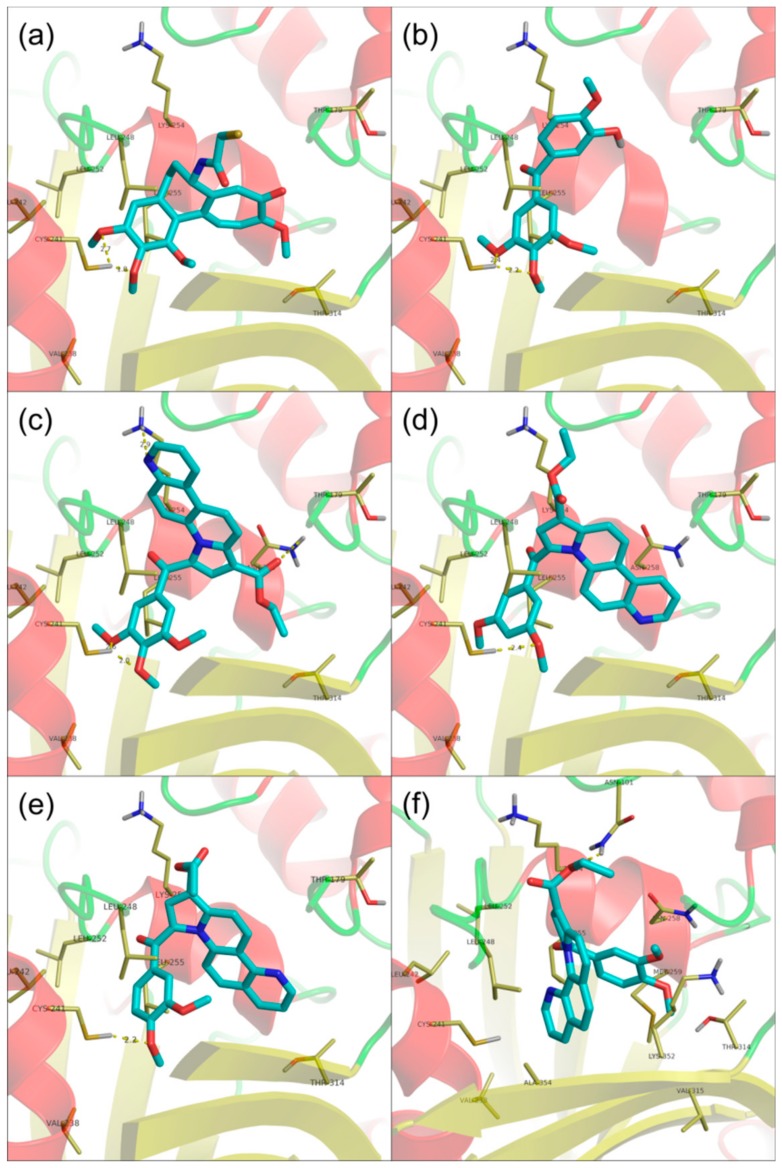
Binding modes of (**a**) colchicine, (**b**) phenstatin, (**c**) Compound **8a**, (**d**) Compound **8b**, (**e**) Compound **5c**, (**f**) Compound **11c** at the colchicine binding site of tubulin; the α,β-tubulin heterodimer is represented as ribbons; amino acids in the binding site are represented as sticks.

Molecular docking of Compounds **8a** and **8b** in the colchicine binding site of tubulin revealed a similar accommodation to previously reported ligands [28], both having ring A overlapping with the trimethoxyphenyl subunit of phenstatin (Figure 2a–d), and interacting with the protein through hydrogen bonding with βCys241. The ligands are further stabilized in the binding pocket through hydrophobic interactions with βLeu242, βLeu248, βAla250 and βLeu255. In addition, Compound **8a** interacts with βLys254 through the N1 nitrogen of the 4,7-phenanthroline subunit, forming a hydrogen bond, and with βAsn258 through its ester functional group, interactions which are absent in the case of analogue **8b**. These two amino acids have been previously identified as key interaction partners for other microtubule depolymerizing agents [34] or inhibitors of tubulin polymerization [35]. Thus, removal of the 4-methoxy subunit in **8b** leads to the loss of one hydrogen bond between the ligand and βCys241, and subsequent inability to interact with βLys254 and βAsn258, which could account for the lack of activity observed for **8b**. Further mutagenesis studies could confirm the involvement of βLys254 and βAsn258 in the observed activity of **8a**.

Compound **5c** is accommodated in a similar fashion to 4,7-phenanthroline analogue **8b**, with ring A overlapping with the trimethoxyphenyl subunit of phenstatin, and forming hydrophobic contacts with βLeu242, βLeu248, βAla250 and βLeu255. In addition, it forms a hydrogen bond with βCys241, but does not interact through other types of contact with tubulin residues (Figure 2e). The lack of stronger electrostatic interactions between this compound and tubulin could account for its reduced antiproliferative effect when compared to active analogue **8a**. Subsequent inhibition binding experiments against colchicine could confirm the accommodation of this compound and analogues **8a** and **8b** in the proposed modes.

Interestingly, the best-scoring pose of 1,10-phenanthroline derivative **11c** is accommodated in the colchicine binding site of tubulin in a different manner, so as to permit a hydrogen bond interaction with αAsn101 (Figure 2f), which has been identified as an important interaction partner for other tubulin polymerization inhibitors which bind to the colchicine binding site [36]. In addition, the pyrrolo[1,2-*a*][1,10]phenanthroline moiety of **11c** is stabilized in the binding pocket through an extensive hydrophobic network formed by βLeu248, βAla354 and βLys352, while the 3,4-dimethoxyphenyl ring is stabilized by contacts with βLeu255, βVal315 and βMet259. Other binding site residues from the α subunit include αThr179, αAla180 and αVal181. It is unclear if the observed superior antiproliferative effects of **11c** compared to 1,7- and 4,7- analogues are due to a different binding mechanism, and therefore further mutagenesis experiments are required.

## 3. Materials and Methods

### 3.1. Chemistry

All of the commercially available reagents and solvents employed were used without further purification. The melting points were recorded on an A. Krüss Optronic Melting Point Meter KSPI and are uncorrected. Analytical thin-layer chromatography was performed with commercial silica gel plates 60 F254 (Merck Darmstadt, Germany) and visualized with UV light (λ_max_= 254 or 365 nm). The NMR spectra were recorded on a (Bruker Vienna, Austria) Avance III 500 MHz spectrometer or a BrukerAvance 400 DRX (400 MHz). Chemical shifts were reported in delta (δ) units, part per million (ppm) and coupling constants (*J*) in Hz. The following abbreviations were used to designate chemical shift multiplicities: s = singlet, d = doublet, t = triplet, q = quartet, m = multiplet, bs = broad singlet. Infrared (IR) data were recorded as films on potassium bromide (KBr) pellets on a FT-IR (Shimadzu Kyoto, Japan) Prestige 8400s spectrophotometer or a Jasco 660 Plus FTIR spectrophotometer. Analyses indicated by the symbols of the elements or functions were within ±0.4% of the theoretical values.

#### 3.1.1. General Procedure for the Synthesis of Monoquaternary Salts **3a**–**d**, **7a**–**d** and **10a**–**d**

1 mmol of heterocycle (1,7-phenanthroline (**1**), 4,7-phenanthroline (**6**) or 1,10-phenanthroline (**9**)) was dissolved in minimum volume of acetone. Then, 1.1 mmol of reactive halide (2-bromo-1-(3,4,5-trimethoxyphenyl)ethanone **2a**, 2-bromo-1-(3,5-dimethoxyphenyl) ethanone **2b**, 2-bromo-1-(3,4-dimethoxyphenyl) ethanone **2c** or 2-bromo-1-(4-bromophenyl) ethanone **2d**) was added and the resulting mixture was stirred overnight at room temperature. The formed precipitate was filtered and washed with diethyl ether to give the desired product which was used in the next reaction without any further purification.

#### 3.1.2. General Procedure for the Preparation of Compounds **5a**–**d**, **8a**–**d** and **11a**–**d**

The cycloimmonium salt (**3**, **7** or **10**) (1 mmol) and ethyl propiolate (1.1 mmol) were added to dichloromethane (DCM) and the obtained suspension was stirred at room temperature. Then, a solution of triethylamine (TEA) (3 mmol) in DCM (3 mL) was added drop-wise over 1 h (magnetic stirring) and the resulting mixture was then stirred overnight at room temperature. Methanol (10 mL) was added and the resulting solid was collected by filtration to give a solid which was washed with 5 mL methanol. The product was then crystallized from dichloromethane/methanol (1:1, *v/v*).

#### 3.1.3. 7-(2-Oxo-2-(3,4,5-trimethoxyphenyl)ethyl)-1,7-phenanthrolin-7-ium Bromide **3a**

Beige powder; yield: 50%; mp 120–122 °C; IR (KBr), ν_max_ 2997, 2915, 1676, 1583, 1416, 1344, 1165, 1124 cm^−1^; ^1^H NMR (500 MHz, DMSO-*d*_6_) δ 3.83 (3H, s, OCH_3_), 3.94 (3H, s, OCH_3_), 7.20 (2H, s, H-15), 7.50 (2H, s, H-18,22), 8.03 (1H, dd, *J* = 8.0, 3.0 Hz, H-3), 8.42 (1H, d, *J* = 9.5 Hz, H-6), 8.54 (1H, dd, *J* = 8.5, 6.0 Hz, H-9), 8.71 (1H, d, *J* = 9.5 Hz, H-5), 8.77 (1H, dd, *J* = 8.0 Hz, H-4), 9.32 (1H, dd, *J* = 4.0, 1.0 Hz, H-2), 9.60 (1H, d, *J* = 5.5 Hz, H-8), 10.40 (1H, d, *J* = 8.0 Hz, H-10); ^13^C NMR (125 MHz, DMSO*-d*_6_) δ 56.4 (2 × OCH_3_), 60.4 (OCH_3_), 63.9 (C-15), 106.5 (C-18, C-22), 117.2 (C-6), 123.7 (C-9), 125.3 (C-3), 126.0 (C-11), 128.7 (C-13), 128.8 (C-17), 136.8 (C-5), 137.4 (C-4), 141.1 (C-12), 143.1 (C-10), 143.2 (C-20), 143.3 (C-14), 149.9 (C-8), 152.6 (C-2), 153.0 (C-19, C-21), 189.6 (C-16); Anal. Calcd. for C_23_H_21_BrN_2_O_4_ C, 58.86; H, 4.51; N, 5.97. Found C, 58.90; H, 4.48; N, 6.00.

#### 3.1.4. 7-(2-(3,5-Dimethoxyphenyl)-2-oxoethyl)-1,7-phenanthrolin-7-ium Bromide **3b**

Beige powder; yield 72%; mp 185–180 °C; IR (KBr), ν_max_ 3069, 2923, 1678, 1591, 1447, 1343, 1300, 1220, 839 cm^−1^; ^1^H NMR (500 MHz, DMSO*-d*_6_) δ 3.88 (6H, s, 2 × OCH_3_), 6.96 (1H, bs, H-20), 7.18 (2H, s, H-15), 7.18 (2H, d, *J* = 2.0 Hz, H-18, H-22), 8.03 (1H, dd, *J* = 8.0, 3.5 Hz, H-3), 8.43 (1H, d, *J* = 10.0 Hz, H-6), 8.52 (1H, dd, *J* = 8.0; 6.0 Hz, H-9), 8.70 (1H, d, *J* = 9.5 Hz, H-5), 8.77 (1H, d, *J* = 7.5 Hz, H-4), 9.30 (1H, d, *J* = 3.0 Hz, H-2), 9.64 (1H, d, *J* = 5.0 Hz, H-8), 10.38 (1H, d, *J* = 8.5 Hz, H-10). ^13^C-NMR, (125 MHz, DMSO*-d*_6_) δ 55.8 (OCH_3_), 64.1 (C-15), 106.5 (C-18, C-22), 106.6 (C-20), 117.3 (C-6), 123.7 (C-9), 125.4 (C-3), 126.1 (C-11), 128.7 (C-13), 135.4 (C-17), 136.8 (C-5), 137.4 (C-4), 141.1 (C-12), 143.1 (C-14), 143.2 (C-10), 150.0 (C-8), 152.6 (C-2), 160.8 (C-19, C-21), 190.6 (C-16); Anal. Calcd. for C_22_H_19_BrN_2_O_3_ C, 60.15; H, 4.36; N, 6.38. Found C, 60.26; H, 4.30, N 6.40.

#### 3.1.5. 7-(2-(3,4-Dimethoxyphenyl)-2-oxoethyl)-1,7-phenanthrolin-7-ium Bromide **3c**

Beige powder, yield 59%; mp 231–235 °C; IR (KBr) ν_max_ 3092, 2930, 1680, 1609, 1582, 1447, 1348, 1271, 1130 cm^−1^; ^1^H NMR (500 MHz, DMSO*-d*_6_) δ 3.87 (3H, s, OCH_3_), 3.94 (3H, s, OCH_3_), 7.09 (2H, s, H-15), 7.29 (1H, d, *J* = 8.5 Hz, H-21), 7.59 (1H, s, H-18), 7.90 (1H, d, *J* = 8.0 Hz, H-22), 8.03 (1H, dd, *J* = 7.5, 4.5 Hz, H-3), 8.40 (1H, d, *J* = 9.5 Hz, H-6), 8.52 (1H, t, *J* = 7.0 Hz, H-9), 8.70 (1H, d, *J* = 9.5 Hz, H-5), 8.76 (1H, d, *J* = 7.5 Hz, H-4), 9.33 (1H, d, *J* = 3.0 Hz, H-2), 9.58 (1H, d, *J* = 4.0 Hz, H-8), 10.40 (1H, d, *J* = 8.5 Hz, H-10); ^13^C NMR (125 MHz, DMSO*-d*_6_) δ 55.8 (OCH_3_), 56.1 (OCH_3_), 63.6 (C-15), 110.7 (C-18), 111.3 (C-21), 117.3 (C-6), 123.7 (C-9), 123.9 (C-22), 125.4 (C-3), 126.0 (C-11), 126.3 (C-17), 128.7 (C-13), 136.8 (C-5), 137.4 (C-4), 141.2 (C-12), 143.1 (C-14), 143.12 (C-10), 148.9 (C-19), 150.0 (C-8), 152.6 (C-2), 154.5 (C-20), 188.9 (C-16); Anal. Calcd. for C_22_H_19_BrN_2_O_3_ C, 60.15; H, 4.36; N, 6.38. Found C, 61.16; H, 4.31; N, 6.42.

#### 3.1.6. 7-(2-(4-Bromophenyl)-2-oxoethyl)-1,7-phenanthrolin-7-iumbromide **3d**

Yield 65%; All physical and spectral data are in agreement to literature [31].

#### 3.1.7. 4-(2-(3,4,5-Trimethoxyphenyl)-2-oxoethyl)-4,7-phenanthrolin-4-ium bromide **7a**

Beige powder; yield 70%; mp 220–222 °C; IR (KBr) ν_max_ 2909, 1674, 1584, 1503, 1346, 1165, 1128 cm^−1^; ^1^H NMR (500 MHz, DMSO*-d*_6_) δ 3.83 (3H, s, OCH_3_), 3.93 (6H, s, 2 × OCH_3_), 7.21 (2H, s, H-15), 7.49 (2H, s, H-18, H-22), 8.06 (1H, dd, *J* = 8.5, 4.0 Hz, H-9), 8.55 (1H, d, *J* = 10.0 Hz, H-6), 8.57 (1H, dd, *J* = 8.5, 6.0 Hz, H-2), 8.66 (1H, d, *J* = 10.0 Hz, H-5), 9.27 (1H, d, *J* = 4.0 Hz, H-10), 9.58 (1H, d, *J* = 6.0 Hz, H-8), 9.60 (1H, d, *J* = 8.5 Hz, H-1), 10.32 (1H, d, *J* = 8.5 Hz, H-3); ^13^C NMR (125 MHz, DMSO-*d*_6_) δ 56.4 (2 × OCH_3_), 60.4 (OCH_3_), 64.1 (C-15), 106.5 (C-18, C-22), 120.3 (C-6), 123.6 (C-2), 123.9 (C-13), 124.6 (C-9), 128.0 (C-14), 128.7 (C-17), 133.0 (C-1), 138.4 (C-5), 139.5 (C-11), 143.2 (C-3), 143.4 (C-20), 146.2 (C-12), 149.2 (C-8), 153.4 (C-1), 153.0 (C-19, C-21), 189.6 (C-16); Anal. Calcd. for C_23_H_21_BrN_2_O_4_ C, 58.86; H, 4.51; N, 5.97. Found C, 58.85; H, 4.58; N, 5.93.

#### 3.1.8. 4-(2-(3,5-Dimethoxyphenyl)-2-oxoethyl)-4,7-phenanthrolin-4-ium Bromide **7b**

Beige powder; yield 57%; mp 240–243 °C; IR (KBr) ν_max_ 3051, 2930, 1692, 1591, 1505, 1354, 1296, 1206, 1157, 837 cm^−1^; ^1^H NMR (500 MHz, DMSO-*d*_6_) δ3.88 (6H, s, 2 × OCH_3_), 6.96 (1H, s, H_20_), 7.21 (2H, s, H-15), 7.23 (2H, s, H-18, H-22), 8.04 (1H, dd, *J* = 8.5, 4.5 Hz, H-9), 8.57–8.64 (3H, m, H-5, H-2, H-6), 9.25 (1H, d, *J* = 3.5 Hz, H-10), 9.60 (1H, d, *J* = 8.5 Hz, H-1), 9.63 (1H, d, *J* = 5.5 Hz, H-8), 10.30 (1H, ad, *J* = 8.5 Hz, H-3); ^13^C NMR (125 MHz, DMSO-*d*_6_) δ55.9 (2 × OCH3), 64.2 (C-15), 106.5 (C-18, C-22, C-20), 120.5 (C-6), 123.5 (C-2), 123.9 (C-14), 124.5 (C-9), 128.0 (C-13), 133.0 (C-8), 135.4 (C-17), 138.3 (C-5), 139.4 (C-11), 143.2 (C-3), 146.2 (C-12), 149.2 (C-1), 153.3 (C-10), 160.8 (C-19, C-21), 190.5 (C-16); Anal. Calcd. for C_22_H_19_BrN_2_O_3_; C, 60.15; H, 4.36; N, 6.38. Found C, 60.14; H, 4.39; N, 6.43.

#### 3.1.9. 4-(2-(3,4-Dimethoxyphenyl)-2-oxoethyl)-4,7-phenanthrolin-4-ium Bromide **7c**

Orange solid; yield 50%; mp 223–226 °C; IR (KBr) ν_max_ 3011, 2918, 1676, 1586, 1518, 1341, 1269, 1161, 1021 cm^−1^; ^1^H NMR (500 MHz, DMSO-*d*_6_) δ 3.87 (3H, s, OCH_3_), 3.94 (3H, s, OCH_3_), 7.11 (2H, s, H-15), 7.29 (1H, d, *J* = 8.5 Hz, H-21), 7.59 (1H, d, *J* = 2.0 Hz, H-18), 7.90 (1H, dd, *J* = 8.5; 4.0 Hz, H-22), 8.06 (1H, dd, *J* = 8.5, 4.5 Hz, H-9), 8.54 (1H, d, *J* = 9.0 Hz, H-6), 8.56 (1H, dd, *J* = 8.5, 6.0 Hz, H-2), 8.65 (1H, d, *J* = 9.5 Hz, H-5), 9.27 (1H, dd, *J* = 4.0; 1.5 Hz, H-10), 9.57 (1H, d, *J* = 5.5 Hz, H-8), 9.60 (1H, d, *J* = 8.0 Hz, H-1), 10.30 (1H, d, *J* = 8.5 Hz, H-3); ^13^C NMR (125 MHz, DMSO-*d*_6_) δ 55.8 (OCH_3_), 56.1 (OCH_3_), 63.8 (C-15), 110.7 (C-18), 111.3 (C-21), 120.5 (C-6), 123.4 (C-2), 123.90 (C-13), 123.92 (C-22), 124.5 (C-9), 126.2 (C-17), 128.0 (C-14), 133.0 (C-1), 138.3 (C-5), 139.5 (C-11), 143.1 (C-3), 143.4 (C-20), 146.2 (C-12), 148.9 (C-8), 149.3 (C-19), 153.4 (C-10), 154.6 (C-20), 188.9 (C-16); Anal. Calcd. for C_22_H_19_BrN_2_O_3_ C,60.15; H, 4.36; N, 6.38. Found C, 60.16; H, 4.39; N, 6.40.

#### 3.1.10. 4-(2-(4-Bromophenyl)-2-oxoethyl)-4,7-phenanthrolin-4-ium Bromide **7d**

Beige powder;yield 94%; mp 253–255 °C; IR(KBr) ν_max_ 3037, 2966, 1703, 1586, 1341, 1223, 1180, 1067, 833 cm^−1^; ^1^H NMR (500 MHz, DMSO-*d*_6_) δ 7.13 (2H, s, H-15), 7.95 (2H, bs, H-19, H-21), 8.09 (3H, bs, H-9, H-18, H-22), 8.56–8.64 (3H, m, H-5, H-2, H-6), 9.27 (1H, bs, H-10), 9.58 (2H, m, H-1, H-8), 10.32 (1H, bs, H-3); ^13^C NMR (125 MHz, DMSO-*d*_6_) δ 64.0 (C-15), 129.0 (C-20), 120.6 (C-6), 123.4 (C-2), 123.9 (C-14), 124.5 (C-9), 128.0 (C-13), 130.6 (C-18, C-22), 132.2 (C-19, C-21), 132.7 (C-17), 133.0 (C-8), 138.3 (C-5), 139.6 (C-11), 143.2 (C-3), 146.2 (C-12), 149.3 (C-1), 153.4 (C-10), 190.1 (C-16); Anal. Calcd. for C_20_H_14_Br_2_N_2_O C, 52.43; H, 3.08; N, 6.11. Found C, 52.47; H, 3.10; N, 6.15.

#### 3.1.11. 1-(2-Oxo-2-(3,4,5-trimethoxyphenyl)ethyl)-1,10-phenanthrolin-1-ium Bromide **10a**

Beige powder; yield 68%; mp 161–164°C; IR(KBr) ν_max_ 2997, 2915, 1676, 1611, 1584, 1416, 1344, 1125, 839 cm^−1^; ^1^H NMR (500 MHz, DMSO-*d*_6_) δ 3.84 (3H, s, OCH_3_), 3.91 (3H, s, OCH_3_), 7.20 (2H, s, H-15), 7.48 (2H, s, H-18, H-22), 7.94 (1H, dd, *J* = 8.5; 4.5 Hz, H-8), 8.50 (2H, s, H-5, H-6), 8.61–8.65 (2H, m, H-7, H-3), 8.78 (1H, dd, *J* = 4.5; 1.0 Hz, H-9), 9.61 (1H, d, *J* = 8.0 Hz, H-4), 9.65 (1H, d, *J* = 5.5 Hz, H-2); ^13^C-NMR (125 MHz, DMSO-*d*_6_) δ 56.4 (OCH_3_), 60.4 (2 × OCH_3_), 69.7 (C-15), 105.80 (C-18, C-22), 124.5 (C-3), 124.8 (C-8), 127.0 (C-5), 129.6 (C-17), 130.7 (C-6), 131.5 (C-11), 132.1 (C-12), 136.3 (C-14), 138.0 (C-9), 138.5 (C-13), 142.6 (C-20), 148.2 (C-4), 149.0 (C-7), 152.0 (C-2), 153.0 (C-19, C-21), 189.7 (C-16); Anal. Calcd. for C_23_H_21_BrN_2_O_4_ C, 58.86; H, 4.51; N, 5.97. Found C, 58.84; H, 4.48; N, 6.00.

#### 3.1.12. 1-(2-(3,5-Dimethoxyphenyl)-2-oxoethyl)-1,10-phenanthrolin-1-ium Bromide **10b**

Beige powder; yield 76%; mp 142–143 °C; IR (KBr) ν_max_ 3026, 2993, 1675, 1585, 1419, 1287, 1248, 1190, 849 cm^−1^; ^1^H NMR (500 MHz, DMSO-*d*_6_) δ 3.88 (6H, s, 2 × OCH_3_), 6.97 (1H, bs, H-20), 7.11 (2H, d, *J* = 2 Hz, H-18, H-22), 7.29 (2H, s, H-15), 7.93 (1H, dd, *J* = 8.0; 4.5 Hz, H-8), 8.49 (2H, s, H-5, H-6), 8.56 (1H, dd, *J* = 8.0; 2.0 Hz, H-7), 8.62 (1H, dd, *J* = 8.0; 6.0 Hz, H-3), 8.79 (1H, dd, *J* = 8.0; 2.0 Hz, H-9), 9.60 (1H, d, *J* = 8.5 Hz, H-4), 9.64 (1H, d, *J* = 5.5 Hz, H-2); ^13^C-NMR (125 MHz DMSO-*d*_6_) δ 55.7 (2 × OCH_3_), 65.5 (C-15), 106.4 (C-18,C-22), 106.7 (C-20), 124.9 (C-3), 125.7 (C-8), 127.1 (C-5), 129.7 (C-17), 130.7 (C-6), 131.5 (C-11), 132.1 (C-12), 135.6 (C-17), 136.3 (C-14), 138.1 (C-9), 138.3 (C-13), 148.2 (C-4), 148.8 (C-7), 152.0 (C-2), 160.8 (C-19, C-21), 189.1 (C-16); Anal. Calcd. for C_22_H_19_BrN_2_O_3_ C, 60.15; H, 4.36; N, 6.38. Found C, 60.08; H, 4.30; N, 6.35.

#### 3.1.13. 1-(2-(3,4-Dimethoxyphenyl)-2-oxoethyl)-1,10-phenanthrolin-1-ium Bromide **10c**

Beige powder; yield 94%; mp 253–255 °C; IR (KBr)ν_max_ 2997, 2915, 1676, 1611, 1584, 1416, 1344, 1125, 839 cm^−1^; ^1^H NMR (500 MHz, DMSO-*d*_6_) δ 3.84 (3H, s, OCH_3_), 3.91 (3H, s, OCH_3_), 7.20 (2H, s, H-15), 7.48 (2H, s, H-18, H-22), 7.94 (1H, dd, *J* = 8.5; 4.5 Hz, H-8), 8.50 (2H, s, H-5, H-6), 8.61–8.65 (2H, m, H-7, H-3), 8.78 (1H, dd, *J* = 8.0; 1.0 Hz, H-9), 9.61 (1H, d, *J* = 8.0 Hz, H-4), 9.65 (1H, d, *J* = 5.5 Hz, H-2); ^13^C NMR (125 MHz, DMSO-*d*_6_) δ 56.4 (OCH_3_), 60.4 (2 × OCH_3_), 69.7 (C-15), 105.80 (C-18, C-22), 124.5 (C-3), 124.8 (C-8), 127.0 (C-5), 129.6 (C-17), 130.7 (C-6), 131.5 (C-11), 132.1 (C-12), 136.3 (C-14), 138.0 (C-9), 138.5 (C-13), 142.6 (C-20), 148.2 (C-4), 149.0 (C-7), 152.0 (C-2), 153.0 (C-19, C-21), 189.7 (C-16); Anal. Calcd. for C_23_H_21_BrN_2_O_4_ C, 58.86; H, 4.51; N, 5.97. Found C, 58.84; H, 4.48; N, 6.00.

#### 3.1.14. 1-(2-(4-Bromophenyl)-2-oxoethyl)-1,10-phenanthrolin-1-ium Bromide **10d**

Beige powder; yield 90%; mp 234–236 °C; IR (KBr) ν_max_ 3022, 2983, 1687, 1580, 1528, 1000 cm^−1^; ^1^H NMR (500 MHz, DMSO-*d*_6_) δ 7.26 (2H, s, H-15), 7.92 (1H, dd, *J* = 8.0; 4.0 Hz, H-8), 7.96 (2H, d, *J* = 8.5 Hz, H-19, H-21), 8.14 (2H, d, *J* = 8.0 Hz, H-18, H-22), 8.47–8.51 (3H, m, H-5, H-6, H-7), 8.63 (1H, dd, *J* = 8.0; 6.5 Hz, H-3), 8.78 (1H, d, *J* = 8.0 Hz, H-9), 9.61 (1H, d, *J* = 8.5 Hz, H-4), 9.63 (1H, d, *J* = 5.5 Hz, H-2) 13C NMR (125 MHz, DMSO-*d*_6_) δ 69.4 (C-15), 124.8 (C-3), 125.6 (C-8), 127.0 (C-5), 128.3 (C-20), 130.2 (C-18, C-22), 130.7 (C-6), 131.5 (C-12), 132.0 (C-11), 133.3 (C-17), 132.4 (C-19, C-21), 136.2 (C-14), 138.1 (C-9), 138.3 (C-13), 148.2 (C-4), 148.8 (C-7), 152.1 (C-2), 189.9 (C-16); Anal. Calcd. for C_20_H_14_Br_2_N_2_O C, 52.43; H, 3.08; N, 6.11. Found C, 52.44; H, 3.10; N, 6.09 [32].

#### 3.1.15. Ethyl 9-(3,4,5-trimethoxybenzoyl)pyrrolo[1,2-*i*][1,7]phenanthroline-7-carboxylate **5a**

Beige solid; yield 88%; mp 256–258 °C; IR (KBr) ν_max_ 2980, 1688, 1620, 1584, 1418, 1460, 1339, 1231, 1220, 1171, 1125, 1086 cm^−1^; ^1^H NMR (500 MHz, CDCl_3_) δ 1.43 (3H, t, *J* = 7.0 Hz, H-20), 3.95 (6H, s, 2 × OMe), 4.01 (3H, s, OMe), 4.42 (2H, q, *J* = 7.0 Hz, H-19), 7.40 (2H, s, H-23, H-27), 7.57 (1H, dd, *J* = 7.5, 4.0 Hz, H-2), 7.81 (1H, s, H-8), 7.92 (1H, d, *J* = 9.5 Hz, H-12), 8.10 (1H, d, *J* = 9.5 Hz, H-11), 8.27 (1H, d, *J* = 8.0 Hz, H-1), 8.59 (1H, d, *J* = 9.5 Hz, H-6), 9.08 (1H, ad, *J* = 2.5 Hz, H-3), 9.27 (1H, d, *J* = 9.5 Hz, H-5); ^13^C NMR (125 MHz, CDCl_3_) δ 14.7 (C-20), 56.6 (2 × OCH_3_), 60.4 (C-19), 61.2 (OCH_3_), 107.5 (C-7), 107.8 (C-23, C-27), 118.3 (C-6), 120.5 (C-11), 122.1 (C-2), 122.9 (C-14), 125.0 (C-5), 125.4 (C-17), 127.6 (C-9), 128.1 (C-12), 130.1 (C-8), 133.5 (C-16), 133.8 (C-22), 136.2 (C-1), 141.2 (C-15), 142.7 (C-25), 145.4 (C-13), 150.6 (C-3), 153.3 (C-24, C-26), 164.2 (C-18), 184.0 (C-21); Anal. Calcd. for C_28_H_24_N_2_O_6_ C, 69.41; H, 4.99; N, 5.78. Found C, 69.44; H, 4.95; N, 5.80.

#### 3.1.16. Ethyl 9-(3,5-dimethoxybenzoyl)pyrrolo[1,2-*i*][1,7]phenanthroline-7-carboxylate **5b**

Yellow powder; yield 72%; mp 242–244 °C; IR(KBr) ν_max_3061,2964, 2837, 1700, 1625, 1588, 1439, 1346, 1219, 1154, 1080 cm^−1^; ^1^H NMR (CDCl_3_, 500 MHz) δ 1.43 (3H, t, *J* = 7.0 Hz, H-20),3.89 (6H, s, 2 × OMe), 4.42 (2H, q, *J* = 7.0 Hz, H-19), 6.77 (1H, bs, H-25), 7.26 (2H, bs, H-23, H-27), 7.58 (1H, dd, *J* = 8.0, 3.5 Hz, H-2), 7.82 (1H, s, H-8), 7.92 (1H, d, *J* = 9.5 Hz, H-12), 8.12 (1H, d, *J* = 9.5 Hz, H-11), 8.27 (1H, d, *J* = 8.0 Hz, H-1), 8.60 (1H, d, *J* = 9.5 Hz, H-6), 9.08 (1H, ad, *J* = 2.5 Hz, H-3), 9.27 (1H, d, *J*= 9.5 Hz, H-5); ^13^C NMR (CDCl_3_,125 MHz) δ 14.7 (C-20), 55.9 (2 × OMe), 60.4 (C-19), 105.5 (C-25), 107.6 (C-7), 108.0 (C-23, C-27), 118.3 (C-6), 120.5 (C-11), 122.1 (C-2), 122.9 (C-14), 125.2 (C-5), 125.4 (C-17), 127.7 (C-9), 128.1 (C-12), 130.8 (C-8), 133.8 (C-16), 136.2 (C-1), 140.5 (C-22), 141.4 (C-15), 145.4 (C-13), 150.6 (C-3), 160.9 (C-24, C-26), 164.2 (C-18), 184.3 (C-21); Anal. Calcd. for C_27_H_22_N_2_O_5_ C, 71.35; H, 4.88; N, 6.16. Found C, 71.30; H, 4.79; N, 6.17.

#### 3.1.17. Ethyl 9-(3,4-dimethoxybenzoyl)pyrrolo[1,2-*i*][1,7]phenanthroline-7-carboxylate **5c**

Yellow solid; yield 50%; mp 208–216 °C; IR (KBr) ν_max_ 3061, 2963, 2922, 1697, 1624, 1541, 1440, 1259, 1236, 1219, 1076, 1022 cm^−1^; ^1^H NMR (500 MHz, CDCl_3_) δ 1.43 (3H, t, *J* = 7.0 Hz, H-20), 4.00 (3H, s, OMe), 4.03 (3H, s, OMe), 4.42 (2H, q, *J* = 7.0 Hz, H-19), 7.02 (1H, d, *J* = 8.0 Hz, H-26), 7.57 (1H, dd, *J* = 8.0, 4.5 Hz, H-2), 7.69 (1H, bs, H-23), 7.77 (1H, s, H-8), 7.83 (1H, d, *J* = 8.5 Hz, H-27), 7.90 (1H, d, *J* = 9.5 Hz, H-12), 8.09 (1H, d, *J* = 9.5 Hz, H-11), 8.26 (1H, d, *J* = 8.0 Hz, H-1), 8.59 (1H, d, *J* = 9.5 Hz, H-6), 9.08 (1H, ad, *J* = 3.0 Hz, H-3), 9.25 (1H, d, *J*= 9.5 Hz, H-5);^13^C NMR (125 MHz, CDCl_3_) δ14.7 (C-20), 56.3 (OMe), 56.4 (OMe), 60.4 (C-19), 107.3 (C-7), 110.2 (C-26), 112.1 (C-23), 118.4 (C-6), 120.5 (C-11), 122.1 (C-2), 122.8 (C-14), 124.7 (C-5), 125.36 (C-17), 125.38 (C-27), 127.7 (C-9), 128.0 (C-12), 129.6 (C-8), 131.2 (C-22), 133.8 (C-16), 136.2 (C-1), 140.9 (C-15), 145.5 (C-13), 149.4 (C-24), 150.5 (C-3), 153.6 (C-25), 164.3 (C-18), 184.1 (C-21); Anal. Calcd. for C_27_H_22_N_2_O_5_ C, 71.35; H, 4.88; N, 6.16. Found C, 71.33; H, 4.77; N, 6.12.

#### 3.1.18. Ethyl 9-(4-bromobenzoyl)pyrrolo[1,2-*i*][1,7]phenanthroline-7-carboxylate **5d**

Yield: 65%. All physical and spectral data are in agreement to the literature [16].

#### 3.1.19. Ethyl 9-(3,4,5-trimethoxybenzoyl)pyrrolo[2,1-*c*][4,7]phenanthroline-7-carboxylate **8a**

Beige powder; yield 88%; mp 280–283 °C; IR (KBr) ν_max_ 2988, 2943, 1703, 1630,1582, 1499, 1427, 1352, 1236, 1169, 1130, 1088 cm^−1^; ^1^H NMR (500 MHz, CDCl_3_) δ1.43 (3H, t, *J* = 7.0 Hz, H-20), 3.96 (6H, s, 2 × OMe), 4.01 (3H, s, OMe), 4.43 (2H, q, *J* = 7.0 Hz, H-19), 7.40 (2H, s, H-23, 27), 7.66 (1H, dd, *J* = 8.5, 4.0 Hz, H-3), 8.25 (2H, s, H-11, H-12), 8.51 (1H, d, *J* = 9.5 Hz, H-6), 8.60 (1H, d, *J* = 9.5 Hz, H-5), 8.93 (1H, d, *J* = 8.5 Hz, H-4), 9.04 (1H, d, *J* = 3.0 Hz, H-2); ^13^C NMR (125 MHz, CDCl_3_) δ14.7 (C-20), 56.6 (2 × OCH_3_), 60.5 (C-19), 61.2 (OCH_3_), 107.7 (C-7), 107.8 (C-23, C-27), 118.2 (C-5), 120.7 (C-14), 122.4 (C-6), 122.8 (C-3), 123.4 (C-11), 125.1 (C-13), 127.8 (C-9), 129.6 (C-8), 130.8 (C-12), 131.3 (C-4), 131.8 (C-16), 133.3 (C-22), 140.2 (C-15), 142.8 (C-25), 150.6 (C-2), 146.4 (C-17), 153.3 (C-24, C-26), 164.2 (C-18), 184.1 (C-21); Anal. Calcd. for C_28_H_24_N_2_O_6_ C, 69.41; H, 4.99; N, 5.78. Found C, 69.43; H, 5,00; N, 5.80.

#### 3.1.20. Ethyl 9-(3,5-dimethoxybenzoyl)pyrrolo[2,1-*c*][4,7]phenanthroline-7-carboxylate **8b**

Beige powder; yield 50%; mp 238–240°C; IR (KBr) ν_max_2986, 1701, 1638, 1591, 1497, 1356, 1244, 1206, 1157, 1086, 1069 cm^−1^; ^1^H NMR (500 MHz, CDCl_3_) δ1.42 (3H, t, *J* = 7.0 Hz, H-20), 3.89 (6H, s, 2 × OMe), 4.41 (2H, q, *J* = 7.0 Hz, H-19), 6.78 (2H, t, *J* = 2.0 Hz, H-25), 7.27 (2H, s, H-23, H-27), 7.65 (1H, dd, *J* = 8.0; 4.0 Hz, H-3), 7.81 (1H, s, H-8), 8.22–8.27 (2H, overlapped signals, H-11, H-12), 8.50 (1H, d, *J* = 9.5 Hz, H-6), 8.60 (1H, d, *J* = 9.5 Hz, H-5), 8.92 (1H, d, *J* = 8.5 Hz, H-4), 9.04 (1H, bs, H-2);^13^C NMR (125 MHz, CDCl_3_) δ14.7 (C-20), 55.9 (2 × OMe), 60.5 (C-19), 105.7 (C-25), 107.7 (C-7), 108.1 (C-23, C-27), 118.7 (C-5), 120.8 (C-14), 122.4 (C-3), 123.0 (C-6), 123.5 (C-11), 125.1 (C-13), 128.0 (C-9), 130.3 (C-8), 130.8 (C-12), 131.2 (C-22), 131.9 (C-4), 140.3 (C-16), 140.5 (C-15), 146.4 (C-17), 150.6 (C-2), 161.0 (C-24, C2-6), 164.1 (C-18), 184.4 (C-21); Anal. Calcd. for C_27_H_22_N_2_O_5_ C, 71.35; H, 4.88; N, 6.16. Found C, 71.34; H, 4.90; N, 6.20.

#### 3.1.21. Ethyl 9-(3,4-dimethoxybenzoyl)pyrrolo[2,1-*c*][4,7]phenanthroline-7-carboxylate **8c**

Beigesolid; yield 50%; mp 261–268°C; IR (KBr) ν_max_ 3078, 2978, 2936, 1697, 1630, 1595, 1495, 1344, 1279, 1239, 1170, 1136, 1080, 1024 cm^−1^; ^1^H NMR (500 MHz, CDCl_3_) δ1.42 (3H, t, *J* = 7.0 Hz, H-20), 4.00 (6H, bs, 2 × OMe), 4.40 (2H, q, *J* = 7.0 Hz, H-19), 7.03 (2H, bs, H-26), 7.62–7.83 (4H, m, H-3, H-27, H-23, H-8), 8.21 (2H, bs, H-11, H-12), 8.44 (1H, bs, H-6), 8.54 (1H, bs, H-5), 8.88 (1H, bs, H-4), 9.00 (1H, bs, H-2);^13^C NMR (125 MHz, CDCl_3_) δ14.7 (C-20), 56.3 (OMe), 56.4 (OMe), 60.4 (C-19), 107.4 (C-7), 110.2 (C-26), 112.1 (C-23), 118.6 (C-5), 120.6 (C-14), 122.3 (C-6), 122.4 (C-3), 123.3 (C-11), 125.0 (C-13), 125.5 (C-27), 127.9 (C-9), 129.2 (C-8), 130.7 (C-12), 130.9 (C-22), 131.2 (C-4), 131.7 (C-16), 139.9 (C-15), 146.3 (C-17), 149.4 (C-24), 150.5 (C-2), 153.7 (C-25), 164.2 (C-18), 184.1 (C-21); Anal. Calcd. for C_27_H_22_N_2_O_5_ C, 71.35; H, 4.88; N, 6.16. Found C, 71.34; H, 4.85; N, 6.19.

#### 3.1.22. Ethyl 9-(4-bromobenzoyl)pyrrolo[2,1-*c*][4,7]phenanthroline-7-carboxylate **8d**

Beige powder; yield 50%; mp 260–263 °C; IR (KBr) ν_max_ 2915, 1722, 1687, 1622, 1391, 1298, 1157, 1111 cm^−1^; ^1^H NMR (500 MHz, CDCl_3_) *δ*1.42 (3H, t, *J* = 7.0 Hz, H-20), 4.41 (2H, q, *J* = 7.0 Hz, H-19), 7.66–7.75 (4H, m, H-24, H-26, H-8, H-3), 8.02 (2H, d, *J* = 7.0 Hz, H-23, H-27), 8.25 (2H, bs, H-11, H-12), 8.53 (1H, d, *J* = 9.5 Hz, H-6), 8.61 (1H, d, *J* = 9.5 Hz, H-5), 8.93 (1H, d, *J* = 8.0 Hz, H-4), 9.04 (1H, bs, H-2);^13^C NMR (125 MHz, CDCl_3_) δ 14.7 (C-20), 60.4 (C-19), 107.9 (C-7), 118.6 (C-5), 120.9 (C-14), 122.5 (C-3), 123.3 (C-6), 123.4 (C-11), 125.1 (C-13), 127.6 (C-25), 128.3 (C-9), 130.4 (C-8), 130.9 (C-12), 131.3 (C-4), 131.7 (C-23, C-27), 131.9 (C-16), 132.1 (C-24, C-26), 137.3 (C-22), 140.6 (C-15), 146.5 (C-17), 150.7 (C-2), 164.1 (C-18), 183.6 (C-21); Anal. Calcd. for C_25_H_17_BrN_2_O_3_ C, 63.44; H, 3.62; N, 5.92. Found C, 63.43; H, 3.60; N, 5.89.

#### 3.1.23. Ethyl 11-(3,4,5-trimethoxybenzoyl)pyrrolo[1,2-*a*][1,10]phenanthroline-9-carboxylate **11a**

Beige powder; yield 29%; mp258–260 °C; IR (KBr) ν_max_ 2985, 1702, 1640, 1590, 1497, 1357, 1242, 1208, 1157, 1085, 1070 cm^−1^; ^1^H NMR (400 MHz, CDCl_3_) δ1.42 (3H, t, *J* = 7.2 Hz, CH_3_), 3.97 (6H, s, 2 × OMe), 4.02 (3H, s, OMe), 4.42 (2H, q, *J* = 7.2 Hz, CH_2_), 7.36 (1H, dd, *J* =7.6; 4.0 Hz, H-9),7.41 (2H, s, H-20, H-24), 7.61 (1H, s, H-2), 7.78 (1H, d, *J* = 8.4 Hz, H-6), 7.88 (1H, d, *J* = 8.4 Hz, H-7), 7.95 (1H, d, *J* = 8.4 Hz, H-5), 8.20 (1H, d, *J* = 8.0 Hz, H-8), 8.42 (1H, d, *J* = 2.4 Hz, H-10), 8.55 (1H, d, *J* =8.8 Hz, H-4); ^13^C NMR (100 MHz, CDCl_3_) δ14.7 (CH_3_), 56.6 (2 × OCH_3_), 60.3 (CH_2_), 61.1 (OCH_3_), 106.5 (C-3), 107.8 (C-20, 24), 120.2 (C-4), 121.5 (C-2), 122.6 (C-9), 124.7 (C-6), 124.8 (C-5), 125.3 (C-1), 127.6 (C-15), 129.6 (C-14), 127.8 (C-7), 133.1 (C-19), 135.9 (C-8), 138.3 (C-17), 138.6 (C-16), 142.7 (C-22), 146.0 (C-10), 152.6 (C-13), 153.4 (C-21, 23), 164.5 (COO), 184.7 (C-18); Anal. Calcd. for C_28_H_24_N_2_O_6_: C, 69.41; H, 4.99; N, 5.78. Found: C, 69.45; H, 5,02; N, 5.80.

#### 3.1.24. Ethyl 11-(3,5-dimethoxybenzoyl)pyrrolo[1,2-*a*][1,10]phenanthroline-9-carboxylate **11b**

Beige powder; yield 25%; mp 261–263 °C; IR (KBr) ν_max_ 2985, 2945, 1700, 1633,1581, 1498, 1429, 1355, 1237, 1168, 1132, 1086 cm^−1^; ^1^H NMR (400 MHz, CDCl_3_) δ 1.43 (3H, t, *J* = 7.2 Hz, CH_3_), 3.90 (3H, s, OCH_3_), 4.42 (2H, q, *J* = 7.2 Hz, CH_2_), 6.80 (1H, bs, H-22), 7.27 (2H, bs, H-20, H-24), 7.35 (1H, dd, *J* = 7.2; 4.0 Hz, H-9), 7.57 (1H, s, H-2), 7.82 (1H, d, *J* = 8.4 Hz, H-6), 7.88 (1H, d, *J* = 8.4 Hz, H-7), 7.96 (1H, d, *J* = 8.4 Hz, H-5), 8.20 (1H, d, *J* = 8.0 Hz, H-8), 8.45 (1H, d, *J* = 2.8 Hz, H-10), 8.55 (1H, d, *J* =8.8 Hz, H-4); ^13^C NMR (100 MHz, CDCl_3_) δ 14.6 (CH_3_), 56.0 (2 × OCH_3_), 60.1 (CH_2_), 106.0 (C-3), 108.3 (C-20, C-24), 120.1 (C-4), 121.6 (C-2), 122.6 (C-9), 124.7 (C-5), 124.8 (C-6), 125.4 (C-1), 127.5 (C-7), 127.9 (C-15), 129.4 (C-14), 130.4 (C-19), 135.8 (C-8), 138.1 (C-17), 138.5 (C-16), 146.1 (C-10), 152.7 (C-13), 160.8 (C-21, C-23), 164.6 (COO), 184.4 (C-18); Anal. Calcd. for C_27_H_22_N_2_O_5_: C, 71.35; H, 4.88; N, 6.16. Found: C, 71.34; H, 4.53; N, 6.19.

#### 3.1.25. Ethyl 11-(3,4-dimethoxybenzoyl)pyrrolo[1,2-*a*][1,10]phenanthroline-9-carboxylate **11c**

Beige solid;yield 50%; mp 266–268 °C; IR (KBr) ν_max_ 2932; 1688; 1595, 1514, 1458, 1415, 1261, 1232, 1140, 1024 cm^−1^; ^1^H NMR (400 MHz, CDCl_3_) δ1.42 (3H, t, *J* = 7.2 Hz, CH_3_), 3.95 (3H, s, OMe), 4.03 (3H, s, OMe), 4.40 (2H, q, *J* = 7.2 Hz, CH_2_), 7.03 (2H, d, *J* = 8.4 Hz, H-23), 7.37 (1H, dd, *J* =7.6; 4.0 Hz, H-9), 7.59 (1H, s, H-2), 7.70–7.74 (2H, m, H-20, H-24), 7.80 (1H, d, *J* = 8.4 Hz, H-6), 7.89 (1H, d, *J* = 8.4 Hz, H-7), 7.98 (1H, d, *J* = 8.4 Hz, H-5), 8.19 (1H, d, *J* = 8.0 Hz, H-8), 8.44 (1H, d, *J* = 2.8 Hz, H-10), 8.58 (1H, d, *J* = 9.2 Hz, H-4); ^13^C NMR (100 MHz, CDCl_3_)δ 14.6 (CH_3_), 56.0 (OMe), 56.1 (OMe), 60.0 (CH_2_), 106.1 (C-3), 110.1 (C-23), 112.2 (C-20), 120.0 (C-4), 121.7 (C-2), 122.5 (C-9), 124.8 (C-5), 125.5 (C-1), 127.7 (C-15), 125.6 (C-24), 124.9 (C-6), 129.6 (C-14), 127.6 (C-7), 130.5 (C-19), 135.8 (C-8), 138.2 (C-17), 138.7 (C-16), 146.2 (C-10), 148.8 (C-21), 152.7 (C-22, C-13), 164.7 (COO), 184.5 (C-18); Anal. Calcd. for C_27_H_22_N_2_O_5_ C, 71.35; H, 4.88; N, 6.16. Found C, 71.33; H, 4.51; N, 6.19.

#### 3.1.26. Ethyl 11-(4-bromobenzoyl)pyrrolo[1,2-*a*][1,10]phenanthroline-9-carboxylate **11d**

Yield 65%; All physical and spectral data are in agreement to the literature [33].

### 3.2. Cell Proliferation Assay

The compounds were tested against a panel of 60 human cancer cell lines at the National Cancer Institute, Rockville, MD. The cytotoxicity experiments were realized using a 48-h exposure protocol using sulphorhodamine B assay [37,38,39].

### 3.3. Molecular Modelling

Flexible docking experiments were carried out as previously reported [28]. Briefly, a 18 × 22 × 22 Å^3^ gridbox was used, centred on the colchicine binding site of the α,β-tubulin heterodimer crystal structure (PDB: 1SA0) [40] and experiments were carried out using Autodock Vina [41]. One hundred poses were generated for each ligand, and the best ranked models were chosen for further visual inspection in order to assess the consistency of the generated docking solutions relative to the docking poses of the known inhibitors colchicine and phenstatin. Molecular graphics and visual analyses were performed in the PyMOL Molecular Graphics System, Version 1.8.2. (Schrödinger, LLC, New York, NY, USA).

## 4. Conclusions

Three new classes of potential phenstatin analogues have been synthesized and characterized. Four compounds were selected by NCI and tested against a panel of 60 cell lines at a single concentration of 10^−5^ M using sulphorhodamine B assay. The results show that the two compounds, **8a** and **11c,** inhibit cell proliferation in several cancer cell lines, the derivative **11c** being the most active and showing cytotoxic activity against the COLO205, MDA-MB-435 and A498 cell lines. Compound **11c** was further tested in the full five-dose assay and exhibited significant antiproliferative activity against 40 cell lines on NCI with GI_50_ values in the range of 0.296–3.78 μM. Docking studies indicate that the tested compounds most likely exert their antiproliferative activity by interacting with specific residues in the colchicine binding site of tubulin, similar to their parent compound, phenstatin, as well as other classes of tubulin polymerization inhibitors. In terms of improving anticancer activity, the substitution of ring B of phenstatin with a pyrrolo[1,2-*a*][1,10]phenanthroline group appears to be the most beneficial, while the replacement with a pyrrolo[1,2-*i*][1,7]phenanthroline is detrimental to the growth inhibition properties against cancer cell lines. We can conclude that our phenstatin analogue types **8** and **11** are amenable for further structural optimization in the development of chemotherapeutic agents.

## Figures and Tables

**Table 1:** Results of the in vitro growth inhibition (GI%) caused by Compounds **5c**, **8a**, **8b** and **11c** against human cancer cell lines in the single-dose assay ^a.^

Cell Type	Compound	5c	8a	8b	11c	Phenstatin
	Cell Line	GI (%) (10^−5^ M) ^a^				
Leukemia	CCRF-CEM	0	37	1	**80**	94
	K-562	8	**87**	14	**85**	91
	SR	12	**86**	24	77	93
	HL-60(TB)	13	**87**	25	**96**	100 ^b,f^
	MOLT-4	22	69	23	66	85
	RPMI-8226	0	37	0	**95**	87
Non-small Cell Lung Cancer	A549/ATCC	3	36	2	63	82
	HOP-92	0	45	16	**80**	48
	HOP-62	8	38	7	70	77
	NCI-H460	0	18	2	**89**	93
	NCI-H522	9	77	19	**99**	88
Colon Cancer	COLO205	0	67	0	**100** ^b,c^	58
	HCT-116	0	78	24	73	96
	HCT-15	8	56	18	69	96
	HT-29	0	**80**	20	**93**	85
	SW-620	8	76	5	**87**	78
	KM12	3	58	11	71	91
CNS Cancer	SF-295	0	37	0	**88**	100 ^b,g^
	SF-539	0	44	4	**91**	100 ^b,h^
	SNB-75	21	35	18	**82**	100 ^b,i^
	U251	0	49	1	75	79
Melanoma	LOX IMVI	4	53	0	68	85
	M14	0	68	19	**92**	100 ^b,j^
	MDA-MB-435	0	**91**	7	**100** ^b,d^	100 ^b,k^
	UACC-62	0	42	0	53	55
	SK-MEL-2	0	59	0	**81**	40
	SK-MEL-5	1	66	1	**83**	100 ^b,l^
Ovarian Cancer	OVCAR-3	0	39	0	**90**	100 ^b,m^
	NCI/ADR-RES	6	66	4	78	100 ^b,n^
	SK-OV-3	16	22	28	**87**	53
	OVCAR-8	9	44	0	71	86
Renal cancer	A498	0	39	0	**100** ^b,e^	25
	RXF393	0	33	0	75	99
	786-0	0	22	0	67	-
Breast cancer	MCF7	19	71	13	78	94
	MDA-MB-468	0	30	0	61	100 ^b,o^
	HS 578T	3	27	4	75	71
	BT-549	0	31	18	67	88
Prostate cancer	PC-3	13	36	18	78	80
	DU-145	0	11	0	78	90

^a^ Data obtained from NCI’s in vitro 60 cell one dose screening at 10^−5^ M concentration; ^b^ Cytotoxic effect; Cell growth percent: ^c^ −2; ^d^ −26;^e^ −5;^f^ −29;^g^ −9;^h^ −22;^I^ −1;^j^ −4;^k^ −41;^l^ −60;^m^−7;^n^ v32; ^o^ −14.

**Table 2:** Results of the five-dose in vitro human cancer cell growth inhibition ^a^ for Compound **11c** and control phenstatin.

Cell Type	Compound→	11c	Phenstatin
	Cell Line↓	GI_50_ (μM) ^b^	
Leukemia	HL-60(TB)	2.78	0.011
	SR	0.807	<0.010
	CCRF-CEM	3.13	0.034
Non-small Cell Lung Cancer	NCI-H460	1.58	0.033
	NCI-H522	0.611	0.027
	HOP-62	3.78	0.073
Colon Cancer	HCT-116	0.619	0.038
	HCT-15	1.25	<0.010
	HT29	1.42	2.95
	SW-620	0.930	<0.010
	KM12	1.30	<0.010
CNS Cancer	SF-295	0.800	0.367
	SF-539	2.10	0.011
	SNB-75	2.04	<0.010
	U251	3.61	0.043
Melanoma	SK-MEL-5	0.836	0.040
	M14	0.648	<0.010
	MDA-MB-435	**0.296**	<0.010
	UACC-62	0.918	0.448
	LOXIMVI	2.60	0.013
	MALME-3M	1.24	-
	SK-MEL-2	2.67	0.520
	SK-MEL-28	3.70	65.20
Ovarian Cancer	OVCAR-3	1.08	0.021
	NCI/ADR-RES	0.948	0.012
	IGROV1	2.33	0.18
	OVCAR-8	3.73	0.042
Renal Cancer	786-0	2.34	0.905
	A498	2.82	2.28
	UO-31	0.891	0.074
	ACHN	2.40	0.042
	RXF 393	2.06	0.016
Breast cancer	MCF7	2.25	0.033
	HS 578T	3.04	0.031
	MDA-MB-231/ATCC	2.66	0.029
	BT-549	1.94	0.034
	T-47D	2.37	30.4
	MDA-MB-468	2.83	2.71
Prostate cancer	PC-3	0.960	0.045
	DU-145	3.44	0.039

^a^ Data obtained from NCI’s in vitro 60 cell five-dose screening. ^b^ GI_50_ – the molar concentration of tested compound causing 50% growth inhibition of tumor cells.
